# Public Awareness of Automated External Defibrillator Locations

**DOI:** 10.1001/jamanetworkopen.2024.38319

**Published:** 2024-10-10

**Authors:** Chien-Tai Huang, Chi-Hsin Chen, Chun-Hsiang Huang, Cheng-Yi Fan, Jiun-Wei Chen, Matthew Huei-Ming Ma, Edward Pei-Chuan Huang

**Affiliations:** 1 Department of Emergency Medicine, National Taiwan University Hospital Hsin-Chu Branch, Hsinchu City, Taiwan; 2 Graduate Institute of Biomedical Informatics, College of Medical Science and Technology, Taipei Medical University, Taipei, Taiwan; 3 Department of Emergency Medicine, National Taiwan University Hospital Yun-Lin Branch, Douliu City, Taiwan; 4 Department of Emergency Medicine, College of Medicine, National Taiwan University, Taipei City, Taiwan; 5 Department of Emergency Medicine, National Taiwan University Hospital, Taipei City, Taiwan

## Abstract

This cross-sectional study examines survey data from a 4-day nationwide telephone survey in Taiwan about awareness of and willingness to use automated external defibrillators.

## Introduction

Timely defibrillation on patients with shockable rhythm during out-of-hospital cardiac arrest significantly increases chances of survival. Despite extensive implementation and educational promotion of automated external defibrillators (AEDs), various studies worldwide still indicate a low rate of public access defibrillation (PAD). A 2013 nationwide survey in Taiwan revealed that although 86.6% of respondents were willing to use an AED on strangers, only 40.6% were aware of the existence of AEDs, and only 37.3% could locate them in public places.^1^ These results suggest that the ability to locate an AED may be key to increasing the usage rate.

Based on previous research, we hypothesized that successful bystander defibrillation requires knowledge, attitude, willingness, and ability to retrieve the nearest AED (Figure).^1,2^ AED retrieval can be divided into passive (guidance from dispatchers or direct device delivery) and active (bystanders retrieving device themselves) approaches.^3,4^ A comprehensive nationwide survey was conducted to examine the public’s perception and willingness to use AEDs.

**Figure.**
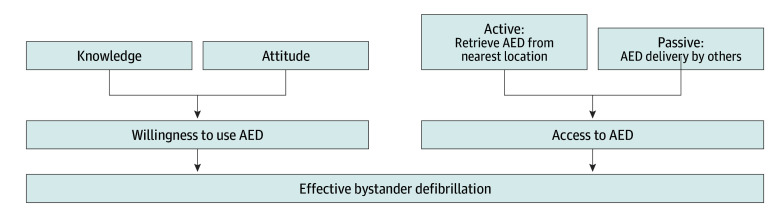
Key Elements to Perform a Successful Bystander Defibrillation A successful bystander defibrillation requires the bystander to possess adequate knowledge of automated external defibrillator (AED) use, a positive attitude toward public access defibrillation, willingness to use an AED, and the ability to access an AED when needed, whether through passive or active means.

## Methods

This cross-sectional, nationwide survey study was conducted by a professional polling agency via telephone and cellphone surveys from April 21 to 24, 2021. Respondents were chosen through stratified random sampling and random-digit dialing. Respondents were asked for their consent. They were told, “We would like to explain and seek your consent: You are free to choose whether or not to participate in this interview.” An 18-item questionnaire obtained demographic data (year of birth, sex, education level, marital status, religious belief, current occupation, medical occupation, personal and family medical history, and whether participant had individuals aged >65 years living with them) and knowledge about and attitude and willingness toward PAD. The questionnaire also investigated public awareness of nearest AED locations from home, on public transportation, and in schools or workplaces (eAppendixes 1 and 2 in Supplement 1). Respondents were asked “Do you have confidence in recognizing an AED (automated external defibrillator) in public places?” Stratified sampling and repeated weighting statistical analysis aligned survey results with national census data. Forced-entry logistic regression was used for multivariable analysis to examine correlation between predictor variables and AED location awareness. *P* < .05 was considered significant. The STROBE reporting guideline was followed. Data were analyzed using IBM SPSS Statistics, version 24. This study was approved by the National Taiwan University Hospital institutional review board.

## Results

A total of 1083 respondents completed the survey. Most respondents (n = 938 [86.6%]) expressed a willingness to perform bystander defibrillation. Among these, 196 of 938 (20.9%) were aware of the AED location near their homes; 141 of 913 (15.4%) were near public transportation, and 225 of 662 (34.0%) were near their school or workplace. Basic demographics of respondents were not associated with increased awareness of AED locations (Table). However, knowledge-related factors showed significant correlations. Respondents confident in recognizing AEDs in public places were significantly more aware of their locations (adjusted odds ratio, 4.48; 95% CI, 1.69-11.89; *P* = .003).

**Table.** Result of the Questionnaire Survey and Factors Associated With Awareness of Nearest AED Location

Questionnaire content	Respondents		Factors associated with awareness of nearest AED location					
			Home		Public transportation		School or workplace	
	Total No.	Those who responded “yes,” No. (%)	aOR (95% CI)	*P* value	aOR (95% CI)	*P* value	aOR (95% CI)	*P* value
Knowledge								
Have you ever heard of AED before?	1083	757 (69.9)	1.71 (0.74-3.98)	.21	0.93 (0.290-3.00)	.91	1.24 (0.45-3.44)	.68
Do you have the confidence in recognizing an AED in public places? (For people who answered “Yes” on question 1)^a^	757	582 (76.9)	3.86 (1.95-7.64)	<.001	4.48 (1.69-11.89)	.003	4.54 (1.90-10.88)	<.001
Do you know how to correctly operate an AED? (For people who answered “yes” on question 1)	757	274 (36.1)	2.3 (1.23-4.28)	.009	1.45 (0.68-3.10)	.34	2.55 (1.24-5.24)	.01
Have you ever participated in an event with the use of AED training course in the past? (For people who answered “yes” on question 1)	757	265 (35.0)	0.77 (0.46-1.29)	.32	0.99 (0.55-1.80)	.98	3.86 (0.93-16.01)	.06
Had received AED training course in recent 2 y (For people who answered “yes” on question 1.3)	265	157 (59.2)	NA	NA	NA	NA	NA	NA
Attitude								
If there are free AED training courses, would you like to attend them? (For respondents who never received AED training course)	818	574 (70.2)	0.93 (0.54-1.60)	.78	1.12 (0.520-2.44)	.77	1.61 (0.77-3.37)	.21
Does the general public need to learn the use of AEDs?^a^	1083	968 (89.4)	NA	NA	1.45 (0.53-3.99)	.47	0.7 (0.30-1.65)	.41
Willingness								
If a stranger collapses and an AED is available, would you be willing to use it?^a^	1083	938 (86.6)	NA	NA	NA	NA	NA	NA
Awareness	NA	NA	NA	NA	NA	NA	NA	NA
Do you know any AED location nearest from your home?	1083	222 (20.5)						
Do you know any AED location nearest from public transportation?^b^	1053	157 (14.9)	NA	NA	NA	NA	NA	NA
Do you know any AED location nearest from school or workplace?^c^	758	259 (34.2)	NA	NA	NA	NA	NA	NA
Sex								
Male	530	530 (49.0)	1.26 (0.82-1.93)	.3	1.37 (0.81-2.31)	.24	NA	NA
Female	553	553 (51.1)	Reference	NA	Reference	NA	NA	NA
Age, y								
20-39	1083	361 (33.4)	NA	.12	NA	.47	NA	.17
40-59 (Reference)	1083	411 (38.0)	0.6 (0.37-0.98)	.04	1.05 (0.56-1.97)	.87	0.64 (0.38-1.07)	.09
≥60	V	310 (28.7)	0.91 (0.49-1.70)	.78	0.6 (0.25-1.44)	.25	0.6 (0.22-1.65)	.32
Education								
High school and above	1083	825 (76.2)	1.87 (0.91-3.84)	.09	2 (0.72-5.61)	.19	1.17 (0.44-3.12)	.76
Marital status								
Married	1083	645 (59.6)	NA	NA	0.91 (0.51-1.65)	.77	NA	NA
Religion								
Religious	1083	888 (82.1)	NA	NA	0.79 (0.42-1.46)	.45	NA	NA
Occupation								
Health care providers	1083	55 (5.1)	0.94 (0.26-3.37)	.92	2.8 (0.78-9.98)	.11	1.58 (0.36-7.21)	.56
Personal medical history								
Heart disease	1083	81 (7.5)	0.39 (0.12-1.28)	.12	1.08 (0.34-3.39)	0.9	NA	NA
Cerebrovascular disease	1083	11 (1.0)	NA	NA	NA	NA	NA	NA
Chronic kidney disease	1083	7 (0.6)	NA	NA	NA	NA	NA	NA
Cancer	1083	24 (2.2)	NA	NA	NA	NA	NA	NA
Organ transplant	1083	2 (0.2)	NA	NA	Invalid value^d^	Invalid value^d^	NA	NA
Cohabiting family members’ medical history								
Heart disease	1083	138 (12.8)	NA	NA	NA	NA	NA	NA
Cerebrovascular disease	1083	45 (4.2)	NA	NA	NA	NA	NA	NA
Chronic kidney disease	1083	38 (3.5)	NA	NA	NA	NA	NA	NA
Cancer	1083	84 (7.8)	1.18 (0.55-2.56)	.67	NA	NA	2.032 (90.84-4.91)	.12
Organ transplant	1083	3 (0.3)	NA	NA	Invalid value^d^	Invalid value^d^	NA	NA
Cohabiting family members’ aged >65 y	1083	458 (42.3)	NA	NA	NA	NA	NA	NA
Residence								
Metropolitan city	1083	750 (69.3)	NA	NA	2.14 (1.09-4.20)	.03	NA	NA

Abbreviations: AED, automated external defibrillator; aOR, adjusted odds ratio; NA, not applicable.

^a^ Some respondents declined to answer this question.

^b^ There were 25 respondents who reported that they have never taken public transportation before, and 5 respondents declined to answer the question.

^c^ There were 323 respondents who reported that they had retired or were not going to the school, and 2 respondents declined to answer the question.

^d^ Refers to the occurrence of extremely large values during multivariable logistic regression, with a *P* value approaching 1.0, likely because of the low number of organ transplant cases.

## Discussion

Our research yielded 2 major findings: most respondents willing to use AEDs on strangers could not locate the device during emergencies, and those confident in recognizing AEDs displayed greater awareness of AED locations. Previous research on AED location has primarily focused on deployment strategies.^5^ Furthermore, studies also discovered innovative approaches, such as dispatcher-assisted AED retrieval or drone delivery, that align more closely with the concept of passive access to the AED.^3,4^ Although inspiring, these methods may require additional time for telephone calls and dispatcher confirmation. Various urban environments and high costs may also hinder device delivery feasibility. In contrast, active AED retrieval, where bystanders know and retrieve the nearest AED themselves, may lead to shorter time to defibrillation during high-stress situations.

Despite potential response bias and inherent limitations of questionnaire surveys, we aim to propose public health policies to enhance AED location awareness. Clear and prominent signage, both visual and auditory, and proactive promotion campaigns may help increase confidence in AED recognition. Knowledge about PAD appears to be closely related to public awareness of nearest AED location, indicating a need for further educational promotion. Simulation-based training, where participants can practice cardiopulmonary resuscitation, call for assistance, locate the nearest AED, and engage in briefing and debriefing sessions, could be integrated into the existing educational framework.^6^ A well-implemented AED deployment strategy, combined with public active awareness of AED locations and effective passive delivery, may address the missing piece in bystander defibrillation.

## Notes

Supplement 1. **eAppendix 1.** Questionnaire Content **eAppendix 2.** Detailed Explanations of the Survey Development

Supplement 2.
Data Sharing Statement
