# Supplementary material for: Public Awareness of Automated External Defibrillator Locations
Source: JAMA Netw Open. 2024 Oct 10;7(10):e2438319. doi: 10.1001/jamanetworkopen.2024.38319 (PMC11581478; doi:10.1001/jamanetworkopen.2024.38319)
Supplement: Supplement 1. — eAppendix 1. Questionnaire Content eAppendix 2. Detailed Explanations of the Survey Development [file jamanetwopen-e2438319-s001.pdf]

Supplemental Online Content

Huang CT, Chen CH, Huang CH, et al. Public awareness of automated external defibrillator locations. *JAMA Netw Open*. 2024;7(10):e2438319. doi:10.1001/jamanetworkopen.2024.38319

eAppendix 1. Questionnaire Content
eAppendix 2. Detailed Explanations of the Survey Development

This supplemental material has been provided by the authors to give readers additional information about their work.

eAppendix 1

Questionnaire Content

- Questionnaire Number: ________
- Interviewer Signature: ________________________
- Interview Date: ________ Month ________ Day
- Interview Source: Telephone/Cellphone Interview

Knowledge, Attitude, Willingness of the Public Regarding automated external defibrillator (AED) use

- Interview Area: Taiwan (including outlying islands, Kinmen, Matsu)

© 2024 Huang CT et al. *JAMA Network Open*.

- Interview Target: Telephone users aged 20 and above
- Sampling Method: Computer-assisted telephone interviewing system for landline and mobile phone sampling
- Interview Tool: Telephone interview
- Commissioned Interview Unit: Mountain Water Public Opinion Research Co., Ltd.
- Survey Time: March to May 2021

Before starting the interview, the interviewer must read the following sentence:

Hello! We are interviewers from [OOOO Public Opinion Survey Company], commissioned by the Ministry of Health and Welfare and the Department of Emergency Medicine at OOOO Hospital to conduct a 'Survey on Public Awareness and use of AEDs' May we take a few minutes of your time for this interview?

*If the respondent agrees, please continue to ask: "May I ask if you are over 20 years old?"

□ Over 20 years old, please continue with the consent form and obtain the respondent's consent.

□ Under 20 years old, please say: "Sorry to bother you, thank you, goodbye."

© 2024 Huang CT et al. *JAMA Network Open*.

*If the respondent does not agree to the interview, please say: "Sorry to bother you, thank you, goodbye."

Consent Form

We would like to explain and seek your consent: You are free to choose whether or not to participate in this interview. If you agree to participate, you can refuse to answer any question or provide any information at any time during the interview, and you can stop the interview at any time. This interview will not cause any physical harm, and we will keep your responses confidential and handle them with care to ensure that your rights are not compromised. If you have any questions about this interview, you can contact the project leader, Dr. ○○○○ from the Department of Emergency Medicine at ○○○○ Hospital (Tel: ○○○○ ext. ○○○○). Do you agree to participate in this interview?

【The interviewer checks agree or disagree】

□ 1 Agree: proceed with the following questions

© 2024 Huang CT et al. *JAMA Network Open*.

□ 0 Disagree: Sorry to bother you, thank you, goodbye.

First Part: Public AED (Automated External Defibrillator)

1. Have you heard of an AED (also known as an automated external defibrillator)?
   - □ (1) Yes (continue to 1a, 1b)
   - □ (2) No (skip to 3)

1a. Do you have confidence in recognizing an AED (Automated External Defibrillator) in public places?

□ (1) Very confident

□ (2) Confident

□ (3) Fair

□ (4) Not confident

© 2024 Huang CT et al. *JAMA Network Open*.

□ (5) Very unconfident

1b. Do you know how to correctly operate an AED?

□ (1) Very clear

□ (2) Clear

□ (3) Fair

□ (4) Not clear

□ (5) Very unclear

2. Have you ever participated in a course on using AEDs?
    - □ (1) Yes (continue to 2a, skip to 4)
    - □ (2) No

2a. How long has it been since you last received AED training?

□ (1) Within 1 year

□ (2) 1-2 years (inclusive)

□ (3) 2-5 years (inclusive)

□ (4) More than 5 years

□ (5) Don't remember

3. If there is a free opportunity to learn, are you willing to participate in AED training?

© 2024 Huang CT et al. *JAMA Network Open*.

- □ (1) Very willing
- □ (2) Willing
- □ (3) Fair
- □ (4) Unwilling (continue to 3a)
- □ (5) Very unwilling (continue to 3a)

3a. What are the reasons for your unwillingness? (You may choose more than one)

□ (1) Too busy, no time

□ (2) Feel it won't be useful

□ (3) Afraid I won't be able to learn

□ (4) Believe that emergency personnel will come to the rescue, so I don't need to learn

□ (5) Other: ______

4. Do you think the general public need to learn to use an AED to help family members or others in need?
   - □ (1) Very necessary
   - □ (2) Necessary
   - □ (3) Fair
   - □ (4) Unnecessary
   - □ (5) Very unnecessary
5. If a stranger collapses and you can operate an AED and one is available, would you be willing to use it on them?

© 2024 Huang CT et al. *JAMA Network Open*.

- □ (1) Very willing
- □ (2) Willing
- □ (3) Fair
- □ (4) Unwilling (continue to 5a)
- □ (5) Very unwilling (continue to 5a)

5a. What are the reasons for your unwillingness? (You may choose more than one)

□ (1) Worried that my skills are not proficient and I won't be able to operate the device

□ (2) Concerned about legal issues and do not want trouble

□ (3) Believe that it makes no difference, waiting for emergency personnel to arrive for rescue

□ (4) Afraid of being injured by the defibrillator

□ (5) Other: ______

© 2024 Huang CT et al. *JAMA Network Open*.

Second Part: Location of AEDs

1. In the event of a sudden suspected cardiac arrest in a public place, who do you think can use an AED for emergency rescue? (Read the options one by one, multiple choices allowed)
    - □ (1) All general public
    - □ (2) Trained public
    - □ (3) On-site staff
    - □ (4) Emergency personnel
2. Do you know where the nearest AED is in your residential area?
    - □ (1) Very clear
    - □ (2) Clear
    - □ (3) Not sure
    - □ (4) Not clear

© 2024 Huang CT et al. *JAMA Network Open*.

- ○ □ (5) Very unclear

3. Do you know the location of AEDs in your most frequently used public transport systems (buses, high-speed rail, MRT)?
    - ○ □ (1) Very clear
    - ○ □ (2) Clear
    - ○ □ (3) Not sure
    - ○ □ (4) Not clear
    - ○ □ (5) Very unclear
4. Do you know where the nearest AED is in your workplace or school?
    - ○ □ (1) Very clear
    - ○ □ (2) Clear
    - ○ □ (3) Not sure
    - ○ □ (4) Not clear
    - ○ □ (5) Very unclear

© 2024 Huang CT et al. *JAMA Network Open*.

Third Part: Good Samaritan Law

1. Have you heard of the Good Samaritan law in the Emergency Medical Services Act regarding providing emergency assistance to strangers?
    - □ (1) Yes (continue to 1a)
    - □ (2) No
    - □ (3) Refuse to answer

    1a. Do you know the contents of the regulations protecting rescuers?

    □ (1) Yes

    □ (2) No

2. The Good Samaritan law is designed to protect individuals who provide emergency aid to strangers from being sued, provided there is no intentional or gross negligence. If such legal protection exists, would you be willing to provide

© 2024 Huang CT et al. *JAMA Network Open*.

the following emergency aid to strangers? (Read options 1-3, multiple choices allowed)

- □ (1) Cardiopulmonary resuscitation (CPR)
- □ (2) AED
- □ (3) None (continue to 2a)

2a. What are the reasons for your unwillingness? (You may choose more than one)

□ (1) Worried that my skills are not proficient and I might cause harm to the patient

□ (2) Concerned about contracting infectious diseases

□ (3) Afraid of being injured by the defibrillator

□ (4) Believe that it makes no difference, waiting for emergency personnel to arrive for rescue

© 2024 Huang CT et al. *JAMA Network Open*.

Fifth Part: Demographic Characteristics

- Basic Information
    1. Year of birth: ________ (ROC calendar)
    2. Gender: □ (1) Male □ (2) Female
    3. Education level:
        - □ (1) Illiterate
        - □ (2) Elementary
        - □ (3) Junior high
        - □ (4) High school/vocational
        - □ (5) College/University
        - □ (6) Graduate school or above
        - □ (7) Refuse to answer
    4. Marital status:
        - □ (1) Single
        - □ (2) Married
        - □ (3) Divorced
        - □ (4) Widowed

© 2024 Huang CT et al. *JAMA Network Open*.

- □ (5) Other_______
- □ (6) Refuse to answer

5. Religious belief:
   - □ (1) Buddhism
   - □ (2) Taoism
   - □ (3) Christianity
   - □ (4) Catholicism
   - □ (5) Folk beliefs
   - □ (6) Other______
   - □ (7) None
   - □ (8) Refuse to answer
6. Current occupation:
   - □ (1) Military, public servants, teachers
   - □ (2) Agriculture, fishery, animal husbandry
   - □ (3) Industry
   - □ (4) Commerce
   - □ (5) Freelancers
   - □ (6) Service industry
   - □ (7) Homemakers
   - □ (8) Retired
   - □ (9) Students
   - □ (10) Other____
   - □ (11) Refuse to answer
7. Are you engaged in medical-related work (doctors, nurses, paramedics, or working in hospitals/clinics)?
   - □ (1) Yes

© 2024 Huang CT et al. *JAMA Network Open*.

- □ (2) No
- □ (3) Refuse to answer

- Personal and Family Medical History

1. Do you have any of the following diseases?

   a. Heart disease: □ (1) Yes □ (2) No □ (3) Refuse to answer

   b. Stroke: □ (1) Yes □ (2) No □ (3) Refuse to answer

   c. Uremia (requiring dialysis) or kidney disease: □ (1) Yes □ (2) No □ (3) Refuse to answer

   d. Cancer: □ (1) Yes □ (2) No □ (3) Refuse to answer

   e. Have you ever had an organ transplant? □ (1) Yes □ (2) No □ (3) Refuse to answer

2. Do any of your cohabiting family members have the following diseases?

   a. Heart disease: □ (1) Yes □ (2) No □ (3) Refuse to answer

   b. Stroke: □ (1) Yes □ (2) No □ (3) Refuse to answer

   c. Uremia (requiring dialysis) or kidney disease: □ (1) Yes □ (2) No □ (3) Refuse to answer

   d. Cancer: □ (1) Yes □ (2) No □ (3) Refuse to answer

   e. Have any of them had an organ transplant?

   □ (1) Yes □ (2) No □ (3) Refuse to answer

© 2024 Huang CT et al. *JAMA Network Open*.

3. Do you have family members aged 65 or above living with you:

□ (1) Yes □ (2) No □

eAppendix 2

Detailed explanations of the survey development

Research Survey Description

Table of Contents

1. Research Design and Methods
2. Implementation Instructions
3. Quality Control of Survey Process
4. Training and Management of Interviewers

© 2024 Huang CT et al. *JAMA Network Open*.

Research Design and Methods

1. Survey Scope and Subjects

The survey covers all 22 counties and cities nationwide. The target respondents are individuals aged 20 and above.

2. Survey Period

The telephone interviews were conducted from April 21, 2021 (Wednesday) to April 24, 2021 (Saturday), totaling four days. The interview time was from 6:30 PM to 10:00 PM.

3. Survey Method and Tools

The survey was conducted through telephone interviews using the Computer Assisted Telephone Interviewing System (CATI). The CATI system displays the questionnaire directly on each interviewer's computer screen. Interviewers, wearing headsets, input responses directly and immediately into the computer during the interview, which are then transmitted and stored in the main server via the internet, reducing the chance of manual input errors.

© 2024 Huang CT et al. *JAMA Network Open*.

4. Valid Samples and Sampling Error

The survey completed 1083 valid contacts with individuals aged 20 and above in Taiwan (541 household landline samples and 542 mobile phone samples). Post-stratification weighting was applied based on gender, age, residential area, and education level to match the population structure. The sampling error is within ±2.98 percentage points at a 95% confidence level.

5. Executing Unit

OOOO Public Opinion Research Co., Ltd.

6. Sampling Design

This survey used both "household landline" and "mobile phone" sampling methods. Household Landline: Based on the population structure, stratified random sampling combined with random-digit dialing (RDD) was used. The sample was based on the Taiwan residential phone directory registered with Chunghwa Telecom, stratified by county and city, with phone numbers selected from residential users and supplemented by randomly generating the last two digits. This ensured that households not listed in the directory had an equal chance of being surveyed, increasing coverage and reducing sampling error. Mobile Phone: Numbers were generated by combining prefixes assigned to telecom operators by the National Communications Commission (NCC) with five randomly generated digits, which were then dialed randomly.

7. Pre-Survey Preparations

Prior to the survey, both household and mobile phone samples were entered into the project database using the CATI system's random sequencing to avoid non-human sampling errors from structured sorting.

Implementation Instructions

1. Telephone Interview Equipment

Besides professional project planners and appropriate survey techniques, a robust and comprehensive survey system is essential to improve efficiency and accuracy. Our company uses an IBM SERVER-class host to store over 7.6 million household phone records. The phone number database is updated regularly to enhance completeness and accuracy. We have 18 supervisor workstations to monitor interview quality, viewing computer screens and listening to interview statuses synchronously. We have 80 interviewer workstations to conduct telephone interviews efficiently, with each interviewer equipped with comfortable, independent booths, headsets, and high-sensitivity microphones.

2. Interview Method

Our company uses the Computer Assisted Telephone Interviewing System (CATI), developed by Yuma Technology Co., featuring automatic dialing, specific area phone databases, and various sampling methods. This system saves time and reduces human

© 2024 Huang CT et al. *JAMA Network Open*.

error. The system supports automatic question skipping, response logic checks, random question order, appointment scheduling, and detailed dialing records, enhancing the accuracy of survey data.

Quality Control of Survey Process

✓ Pre-Survey Preparations

1. Project Meetings: Before the survey, a project meeting was held to ensure that all researchers and supervisors understood the survey's objectives, questionnaire structure, and content.
2. Interviewer Training: Interviewers received 3.5 hours of training, covering CATI system operation, interview techniques, and questionnaire content. Practical online interviews were conducted to familiarize interviewers with procedures before formal interviews began.

✓ Interview Monitoring

1. Online Monitoring: Supervisors monitored the interview process in real-time using the CATI system, correcting any deviations from standard procedures immediately.
2. Walking Management: Supervisors provided assistance and resolved issues on-site, ensuring the accuracy of interview methods and procedures.

© 2024 Huang CT et al. *JAMA Network Open*.

✓ Reducing Refusal and Improving Interview Quality

To address potential refusal due to frequent telemarketing and scam calls, measures were taken:

1. Avoiding Refusal: Displaying caller ID, providing verification contacts, and clearly stating the survey's commissioning unit helped reduce refusal rates.
2. Improving Response Quality: Interviewers used standard reassurances to explain the importance and confidentiality of the survey data, ensuring the survey's accuracy.

Quality Control During the Survey

1. Daily Execution Records: Supervisors documented daily execution statuses, highlighting common mistakes and any special respondent feedback for timely improvement.
2. Execution Review Meetings: Based on daily records, project managers held review meetings to improve the survey process's quality.

© 2024 Huang CT et al. *JAMA Network Open*.

Training and Management of Interviewers

Our interviewers are proficient in both Mandarin and Taiwanese, with 5 of them also fluent in Hakka. Many had experience in customer satisfaction surveys, ensuring precision and politeness during interviews.

✓ Interviewer Recruitment and Management:

1. Training: Recruitments undergo a 3.5-hour training course, including CATI operation, interview techniques, and hands-on practice. Only those who pass the online test qualify as formal interviewers.
2. Performance Evaluation: Interview quality and performance are evaluated during each survey, with monthly performance reviews and training based on results.
3. Scheduling: Our interviewers are long-term, reflecting stable interview quality.
4. Emotional Management: Interviewers are trained in self-management of emotions, with supervisors providing support and maintaining a consensus on overall interview quality.

© 2024 Huang CT et al. *JAMA Network Open*.
