# Supplementary material for: Public Awareness of Automated External Defibrillator Locations
Source: JAMA Netw Open. 2024 Oct 10;7(10):e2438319. doi: 10.1001/jamanetworkopen.2024.38319 (PMC11581478; doi:10.1001/jamanetworkopen.2024.38319)
Supplement: Supplement 2. — Data Sharing Statement [file jamanetwopen-e2438319-s002.pdf]

Data Sharing Statement

Huang. Public Awareness of Automated External Defibrillator Locations. *JAMA Netw Open*. Published October 10, 2024. doi:10.1001/jamanetworkopen.2024.38319

Data
Data available: No

Additional Information
Explanation for why data not available: The co-authors did not approve data sharing.
